# Consistency of Using an Auditory Prosthesis Device Post a Sequentially Implanted Cochlear Implant: Data-Logging Evidence

**DOI:** 10.7759/cureus.13370

**Published:** 2021-02-16

**Authors:** Saad Alenzi, Fida Almuhawas, Roa Halawani, Abdulrahman Sanosi

**Affiliations:** 1 Otolaryngology, Neurotology, and Skull Base Surgery, King Abdullah Ear Specialist Center (KAESC) King Saud University, Riyadh, SAU; 2 Otorhinolaryngology - Head and Neck Surgery, King Abdullah Ear Specialist Centre (KAESC) King Abdulaziz University Hospital, King Saud University, Riyadh, SAU; 3 Otolaryngology - Head and Neck Surgery, Skull Base Surgery, Ohud Hospital, Ministry of Health, Al Madinah Al Munawarah, SAU; 4 Otolaryngology, Neurotology, and Skull Base Surgery, King Saud University, Riyadh, SAU; 5 Otolaryngology, Intermountain Healthcare, Salt Lake City, USA

**Keywords:** otology, cochlear implants, data logging

## Abstract

Objectives

The aim of this study was to explore: 1) the average use of each device in sequentially implanted cochlear implants; 2) whether the inter-implant duration between implants produced any significant difference in the average use of the second implant; and 3) whether wearing hearing aids before the implantation of the second cochlear implant affects its average use.

Materials and methods

The study included 20 participants with bilateral Nucleus 24 implants (Cochlear Corporation, Lone Tree, CO). Data regarding various variables were extracted and then analyzed with IBM SPSS Statistics for Mac, version 23 (IBM Corp., Armonk, NY).

Results

The pediatric group included 14 subjects (average age 7.5 years) while the adult group comprised six subjects (average age 37.5 years). The average use of the second device was 0.9 hours per day more than the first in the pediatric group while it was 1.22 hours per day more in the adult group. We also divided the subjects on the basis of duration between the first and second devices and calculated the average use of each device by them. There was no significant difference (p>0.05). The average use by subjects who did and did not use hearing aids before implantation was also insignificant (p>0.05).

Conclusions

No significant difference between the average use of the first and second implants, between the inter-implant duration of the first and second implants, the average use of the second implant, and between using hearing aids before the implantation of the second device and the average use was observed.

## Introduction

A cochlear implant (CI) is a neuroprosthetic device that is surgically implanted in a person with sensorineural hearing loss of moderate to severe range. The basic mechanism of action is replacing the normal hearing process with electrical signals that stimulate the cochlear part of the eighth nerve directly. There are two main components of the device, i.e., the sound processor that is worn externally behind the ear and the inner component that is placed under the skin. The sound processor works by using: one or more microphones that detect sound, a speech processor that selectively filters audible sound, and a transmitter that digitizes and then converts sound into electrical signals. These signals are then transmitted across the skin to the inner component using a radiofrequency transmission. The inner component is made up of two parts: a receiver, which catches the radio signals from the sound processor and transforms them into electrical impulses, and an electrode embedded in the cochlea that stimulates the auditory nerve.

The CI was introduced more than four decades ago and has become the go-to option when hearing aids do not provide sufficient benefit. Speech perception after CI device installation has steadily increased since the early days in the 70s and 80s. Postimplantation, most of the users develop significantly better hearing and speech perception skills, especially when combined with lip reading [1]. The development of atraumatic implantation techniques along with electro-acoustic CI systems has improved hearing significantly in patients who demonstrate residual hearing only in the low-frequency range. Further evolution has led to the adoption of assistive listening devices and algorithms that enhance signals. Such improvements have increased the efficiency of CI devices exponentially since their introduction [2].

An essential factor in the long-term benefit of Cochlear Implants is a process called binaural hearing. It is the mechanism by which the central nervous system parses the auditory signals delivered by the cochleae into auditory objects. The brain creates this illusion of 3D localization and depth by merging the signals coming from both ears. This is called stereophony and is very similar to stereoscopy through which the central nervous system (CNS) makes use of the two eyes to create a visual sense of 3D depth [3]. These modalities help the mind create virtual objects that are uncomplicated to identify and segregate much better than what would be achieved by the use of a single signal from only one side. A simple example is when a sound signal coming from a source located on the left side of a person reaches the right ear later than the left ear because it has to travel further. After experiencing the head-shadow effect, the signal reaches the right ear at a lower intensity. The auditory signals reaching the brain are different in such a case, and this is the inter-aural difference. The brain has two particular tasks after it receives the signal: detect the particular pattern in the inputs from both ears to a definite, single object and detect the differences from the action potentials to place the object in a 3D map of the brain.

In cases of asymmetric unilateral hearing loss, the ability to discriminate sounds lessens significantly when compared to cases of bilateral hearing loss [3]. For most patients, bilateral CIs are superior to unilateral implants [4-5]. Improved sound localization, ease of listening, quality of sound, and speech perception are some of the potential benefits reported by the use of bilateral CI devices. Therefore, it becomes necessary for adults and children suffering from bilateral profound sensorineural hearing loss who receive unilateral CI to have a sequential second cochlear implant. However, data suggest that the duration between the first and second implants in sequential implants is crucial and could become a deciding factor in determining the long-term outcome [6].

In children, exposure to spoken communication is essential because it helps them develop their own language and speech skills. It has been reported that children need to hear approximately 21,000 words every day in order to develop their verbal skills at a satisfactory rate [7]. According to the studies, despite a significant amount of variability in individuals, it was found that the pace of language development after CI device placement far exceeded the language development in deaf children who were not given CIs [8]. Therefore, especially in hearing-impaired children, the regular usage of CIs is needed for better outcomes because hearing is important for the development of language and speech [9].

Many factors, including the age of implantation, presence of other disabilities, development of the inner ear and its malformations, duration of daily use of the implant, and family support and care decide the long-term regular use of the implants [10]. However, special attention has been given to the age at which implantation of CI devices is done. A concept of the ‘critical age’ of when to implant the sequential CI in the second ear in children has been suggested. It recommended that the practice of saving the second ear for implantation at a later age should cease and bilateral implantation in children suffering sensorineural hearing loss should be provided at an early age [11].

To support the concept of ‘critical age,’ objective studies are needed in order to determine if the use of the second implant is significantly lower than the first implant. According to the limited research done by using objective methods to determine the use of CIs, moderate to strong correlations were found between word discrimination scores and CI-related variables, such as time-on speech and time-on air, and patient-related variables such as CI duration and implantation age [12]. However, the lack of more objective data on the usage of sequentially placed implants makes it difficult to prove if both ears are used equally after the procedure.

Therefore, the purpose of this study is to objectively determine the amount of device usage of CIs and determine if patients with specific characteristics have a preference in using one implant more than the other in the case of sequentially placed bilateral CIs.

## Materials and methods

Patients whose implants were performed at the King Abdullah Ear Specialist Center, Riyadh, Saudi Arabia were analyzed retrospectively. The data were acquired from the database of the Cochlear Implant Program, which took place at our facility between April 2013 and July 2018. The inclusion criteria included patients who were sequentially implanted, in which both CI devices had Nucleus 24 multichannel devices (Cochlear Corporation, Lone Tree, CO), and used Cochlear’s Nucleus (N6) sound processor as the data-logging software. All patients who were implanted devices with older version processors and who received an upgrade at any time after the first implant were excluded from the study. Also excluded were the patients who were diagnosed with neurodegenerative problems, such as motor neuron diseases, autism, etc., and those patients who had radiological evidence of inner ear structural malformations.

A programming software developed by Cochlear Ltd. (NSW, Australia) for CI devices, called Custom Sound, was used to export data log files. Each data log file corresponded to one ear and was used to determine the average hours per day of use from the device.

Data from 20 subjects are included in the study and different factors, such as demography, daily usage of CI for both the devices, the time period between the first and second implants, and the use of a hearing aid (HA) in the second ear before implantation was obtained. IBM Statistical Package for the Social Sciences (SPSS) Statistics for Mac, version 23 (IBM Corp., Armonk, NY) was then used to analyze the data, and comparisons were drawn between the pediatric and adult age groups and the first and second CI devices.

Subjects

Of the 20 subjects, 11 were males and nine were females. The subjects’ ages ranged from three to 68 years. Those below the age of 18 were placed in the pediatric age group and included 14 subjects (average age 7.5 years). The rest who were 18 and above were placed in the adult group and included six subjects (average age 37.5 years).

Twelve subjects in the pediatric group were suffering from profound hearing loss in the second ear before implantation. Similarly, four subjects in the adult category were suffering from severe to profound hearing loss in the second ear before implantation. The status of hearing in the second ear before implantation with respect to the pediatric and adult age groups is given in Table 1.

**Table 1:** Status of hearing in the second ear before implantation with respect to the pediatric and adult age groups

			Groups	
			pediatric	Adult
Status of hearing in the 2nd ear before implantation	Severe		1 (5%)	0
	Severe to profound		1 (5%)	4 (20%)
	Profound		12 (60%)	2(10%)
Total			14	6

## Results

The average use of the first implant (C1) in the pediatric age group was 8.77 hours per day while in the second implant (C2), it was 9.67 hours per day. For the adult group, the average use was found to be 10.85 hours per day in C1 and 12.07 hours per day in C2. The second device was on average used 0.9 hours per day more than the first in the pediatric group while it was 1.22 hours per day more in the adult group. No significant difference between the usage of C1 and C2 was found for both age groups. The average hours per day use of C1 and C2 with respect to both age groups are given in Table 2.

**Table 2:** Average hours per day use of C1 and C2 in pediatric and adult groups

Age		C1 (hours per day)	C2 (hours per day)	P =
Pediatric	Mean	8.778214	9.679714	
	N	14	14	0.3296
	Std. Deviation	2.2577364	2.5355239	
Adult	Mean	10.851333	12.072333	
	N	6	6	0.4224
	Std. Deviation	2.8712592	2.1309578	
Total	Mean	9.400150	10.397500	
	N	20	20	0.2317
	Std. Deviation	2.5704532	2.6189973	

The average duration between the implantation of the first and second CI devices was 13.57 months in the pediatric age group and 16.16 months in the adult age group, and the difference between the two was not significant (p = 0.5213). These findings are detailed below in Table 3.

**Table 3:** Months of inter-implant duration in pediatric and adult groups

Groups		N	Mean (months)	Std. Deviation	p-value
Duration between implantation of C1 and C2	Pediatric	14	13.57	7.94	0.5213
	Adult	6	16.16	8.61	

Subjects were also divided into groups on the basis of duration between first and second CI device implantation into categories of zero to six months (five subjects), seven to 12 months (three subjects), 13 - 18 months (four subjects), and more than 19 months (eight subjects). The average hours per day use of C1 and C2 with respect to inter-implant duration is given in Table 3. No significant differences were found between the four categories of duration of implantation for C1 and C2 and the average daily use of C1 and C2 in either age group are given in Table 4.

**Table 4:** Relationship between implant use and inter-implant duration

Age	Duration between C1 and C2 (months)		C1 (hours per day)	C2 (hours per day)	P =
Pediatric	0-6 months	Mean	8.036000	8.484500	
		N	4	4	
		Std. Deviation	1.9390420	2.9709900	0.8088
	7-12 months	Mean	7.377500	9.092500	
		N	2	2	
		Std. Deviation	.5480078	3.6140228	0.5752
	13-18 months	Mean	8.705500	11.327500	
		N	4	4	
		Std. Deviation	1.8875292	1.7992475	0.0910
	19 months and more	Mean	10.293500	9.520750	
		N	4	4	
		Std. Deviation	3.0910923	2.3774229	0.7056
Adult	0-6 months	Mean	12.849000	10.953000	
		N	1	1	
		Std. Deviation	.	.	-
	7-12 months	Mean	13.759000	15.068000	
		N	1	1	
		Std. Deviation	.	.	-
	13-18 months	Mean N Std. Deviation	-	-	-
	19 months and more	Mean	9.625000	11.603250	
		N	4	4	
		Std. Deviation	2.7543829	1.9661996	0.2867

Six subjects (30% of total) in the pediatric age group and all six subjects (30% of total) in the adult age group regularly used hearing aids in the second ear regularly before implantation of the second device. Usage of hearing aids in the non-implanted ear before the second CI device in pediatric and adult groups is given in Table 5

**Table 5:** Usage of hearing aids for non-implanted ear before the second CI device in pediatric and adult age groups

		Age Group		Total
		Pediatric	Adult	
Usage of hearing aids in the 2^nd^ ear before implants	No	3	0	3
	Yes	6	6	12
	Yes, but not regular	5	0	5
Total		14	6	20

No significant difference was found between the average use of C1 and C2 in subjects who used or did not use any hearing aid in the non-implanted ear before receiving the second CI device. The detailed data are given below in Table 6.

**Table 6:** Average use of C1 and C2 with respect to the usage of hearing aids for a non-implanted ear before the second CI device in pediatric and adult age groups

Age	Usage of Hearing Aids in the 2^nd^ ear before implants		C1 (hours per day)	C2 (hours per day)	p =
Pediatric	No	Mean	8.228000	7.610333	
		N	3	3	0.6977
		Std. Deviation	2.2939632	1.1411737	
	Yes	Mean	9.152000	9.707667	
		N	6	6	0.7091
		Std. Deviation	2.3452418	2.6596524	
	Yes, but not regular	Mean	8.659800	10.887800	
		N	5	5	0.2039
		Std. Deviation	2.5702100	2.5225529	
Adult	No		-	-	-
	Yes	Mean	10.851333	12.072333	
		N	6	6	0.4224
		Std. Deviation	2.8712592	2.1309578	
	Yes, but not regular		-	-	-

## Discussion

This study has made use of data logging to objectively measure the average daily use of sequentially placed bilateral CIs and has used the average use as a function of the performance of the two devices. The key findings of the study are that even though there were no significant differences, the subjects used the second cochlear implant for more hours per day, on average, than the first implant. This finding is in contradiction with the results of a study by Sparreboom et al. conducted on children who received sequentially implanted bilateral CIs [13]. According to their findings, irrespective of age at implant of the second CI, the subjects used the second implant significantly less than the first implant. They also reported that during the rehabilitation period, the second implant caused more difficulties in wearing for the children than the first implant. The reason for this finding, according to the researchers, was the dominant use of the first implant.

As mentioned earlier, most of the studies done on the use of the second implant in sequential bilateral CIs did not use objective methods to assess the average use. Sparreboom et al. also used the subjective techniques of interviews and questionnaires filled out by patients and their parents. Therefore, the findings of that study do not objectively demonstrate less use of the second cochlear device.

Results from another study also predicted that patients were more likely not to use the second implant if the new implant did not become comparable with the first one [14]. Results from that research paper showed that on average, speech perception was 28% better in C1 than C2 in group A and 20 percent better in group B. The study also revealed that five years after the surgery for the second implant, 25% of subjects in group A and 10% in group B did not wear the second cochlear implant anymore.

Although the findings of the study are based on objective measurement of speech perception from both the ears after implantation, the technique does not directly measure the daily use of the second cochlear device, and subjective data from the patients was used to reach the conclusion of less daily use.

The second main finding of our study was that there were no significant differences between groups divided on the basis of the duration between the first and second CI device implantation.

Researchers have proposed the concept of a critical period, according to which surgery for the second CI should be performed within a limited time window to maximize its benefits [11]. This term was introduced because of the discovery that without proper stimulus, children do not develop correct hearing and speech functions, especially in the case of congenitally deaf children [9], bilateral implants should be performed as soon as possible.

A study by Illg et al. also reported a statistically significant difference between speech test performance of the first and second implanted ears [15]. This difference depended on the inter-implant duration, and they found a significant correlation between the two factors. The researchers proposed that an interval of up to four years was acceptable in younger children. They also suggested the inter-implant duration for the older children should be even less than the four-years proposed in younger ones.

According to our study, the inter-implant duration does not affect the average use of the second implant. As the average use is taken as a function of performance, according to our study, the inter-implant duration does not affect the average use of the second implant (DM1).

The final finding of our study is that the use of hearing aids in the period between the first and second implants did not show any significant difference in the average use of the second cochlear device after implantation. There are conflicting data found in research papers regarding this correlation, and it is difficult to estimate if the use of hearing aids before implantation improves the use and function of the second cochlear device. According to the findings of Almeida et al., the subjects who used hearing aids before the second cochlear device reported significantly better speech perception when they were tested after implantation of the second CI, both in silent and noisy conditions [16].

In another study, it was reported that the subjects who used hearing aids before the second implant had no significant improvement in speech perception than the subjects who did not use a hearing aid [17]. The observations of this study corroborate the findings of our research. However, more research should be conducted to determine if the use of hearing aids before implantation affects the function and performance of the second cochlear implant significantly.

The main strengths of our study are the cohort design that helped in testing multiple hypotheses and the use of objective methods used to assess the average use of both CIs. However, some limitations should be noted. It includes small sample size and the non-availability of audiological or speech discrimination data.

## Conclusions

From the results of our study, we can conclude that the average use of the second CI device is more than the first implant. There was no significant difference found in either age group; however, the use of the second cochlear implant was on average 0.9 hours more per day than the first CI. Similarly, the adult group on average used the second implant 1.22 hours per day more than the first. We found no significant difference between the inter-implant duration of the first and second implants and the average use of the second implant by either age group. The difference between the use of C1 and C2 by those who used hearing aids and those who did not use hearing aids in the non-implanted ear before implantation of the second device was also insignificant. Further study is indicated to expand the sample size and to include audiologic data.
